# Identifying the Health Concerns of Pregnant British Pakistani Women Living in Deprived Areas: A Qualitative Study

**DOI:** 10.1007/s10995-023-03797-z

**Published:** 2023-10-30

**Authors:** Halima Iqbal, Jane West, Rosemary R. C. McEachan, Melanie Haith-Cooper

**Affiliations:** 1 Faculty of Health Studies, University of Bradford, Bradford, UK; 2 Bradford Institute for Health Research, Bradford Teaching Hospital NHS Foundation Trust, Bradford, UK

**Keywords:** British Pakistani women, Pregnancy, Health concerns, Health inequalities, Deprived areas

## Abstract

**Introduction:**

Pregnant British Pakistani women have disproportionately poorer health than the wider population. Bradford has a strong Pakistani presence and a wide range of public health problems including high levels of gestational diabetes, high obesity rates and a high infant mortality rate, which is highest for babies of Pakistani origin. For women to be healthy, we need to know what concerns they have about their health so they can be addressed appropriately. The aim of this study, therefore, was to explore the health concerns of pregnant British Pakistani women living in deprived areas.

**Methods:**

Semi-structured qualitative interviews were conducted with 21 pregnant Pakistani women in a hospital setting. Data were analysed using thematic analysis.

**Results:**

Pakistani women identified safety issues, barriers to undertaking physical activity in the areas where they live, concerns surrounding exercising during pregnancy and cultural and religious constraints that prevented them from engaging in physical activity. They reported issues around food, concerns around a lack of culturally appropriate diet information, the cost of unhealthy food locally, and the lack of healthy food options in their residences. Women were unsure on where to obtain health promotion information and reported a lack of access in obtaining that information. Language barriers in accessing health promotion information were further reported as a concern.

**Discussion:**

Researchers, midwives, health providers, local authority and policy makers interested in improving the health of pregnant Pakistani women may use these findings to develop further research and interventions to improve the poor health of this population.

## Significance

*What is already known on this subject?* South Asian women have previously identified issues relating to safety in physical activity and cultural barriers to engaging in physical activity but there has been little investigation into the health concern of pregnant Pakistani women.

*What this study adds?* We now have a clearer understanding of the barriers faced by pregnant Pakistani women living in deprived areas when trying to live a healthy lifestyle. This understanding will contribute to the development of strategies for promoting health and improving the outcomes for this population.

## Introduction

British Pakistani women experience poorer health compared to the wider population. This includes breast and cervical cancer (Anderson de Cuevas et al., 2018), depression in pregnancy (Insan et al., 2022), and disproportionately high rates of obesity including in pregnancy (Garcia et al., 2017). Evidence suggests that first trimester obesity is more likely in British Pakistani women than white British women (Heslehurst et al., 2010), leading to obesity related complications including gestational diabetes at a lower body mass index that white British women (Ruhstaller et al., 2017). Maternal diet is a critical lifestyle-related factor that influences foetal development and health in early life (Jama et al., 2022).

Bradford, a city in Northern England, includes the largest proportion of people of Pakistani ethnic origin in the United Kingdom, at 20% (BDMC, 2017). Nearly half of babies born in Bradford have parents of Pakistani origin (Wright et al., 2013). There is exceptionally poor health in Bradford including high obesity rates in adults and children in this population (Santorelli et al., 2022), especially in more deprived areas. Pakistani infants in Bradford have a lower body weight yet more body fat than white British infants which is partially attributed to poor maternal nutrition throughout pregnancy (West et al., 2018). This low birth weight increases their risk of conditions including depression (Insan et al., 2022) and type 2 diabetes in later life and in women, higher than average rates of gestational diabetes (Santorelli et al., 2022). The risk of developing such conditions can be reduced by making lifestyle adaptations.

To support Pakistani women adopting a healthy lifestyle ready for pregnancy, it is important to understand their health concerns. This can enable the development of appropriate strategies to improve their health outcomes. Therefore, the aim of this study was to ascertain the health concerns of pregnant Pakistani women living in deprived inner-city areas of Bradford.

## Methods

### Theoretical Approach

To enable an exploration of women’s health concerns, a qualitative research design underpinned by feminist standpoint theory guided this study. This theory prioritizes thinking from women's or marginalized lives and considers these lives as privileged sites of knowledge production (Van der Tuin, 2016). Therefore, health concerns can be brought to the fore that could not have been otherwise anticipated. To reduce bias during the research process, patient and public involvement (PPI) was undertaken through consultations with experts by experience, who were women that reflected the target population. The following sections will include details about the input from experts by experience.

### Sampling and Recruitment

The study reported in this paper has been performed in accordance with the ethical standards laid down in the 1964 Declaration of Helsinki and its later amendments. The COREQ criteria for reporting qualitative research was followed. A purposive sub-sample of the Born in Bradford Better Start (BIBBS) cohort was selected. The BIBBS birth cohort study commenced in 2016 and recruited mothers and their partners from three of the most deprived wards in Bradford, where large numbers of Pakistani women live. For more information, see Dickerson et al. (2016). As part of the BIBBS study, women were recruited at a routine appointment while awaiting their glucose tolerance test, which is offered to all women in Bradford between 26–28 weeks gestation to diagnose gestational diabetes. NHS ethical approval had already been granted for the BIBBS project and a minor amendment was approved for the current study (Ref no: 15/YH/0455). English speaking and reading (due to time constraints) Pakistani women who were over the age of 18 were approached. and given an information sheet which was also explained to them by HI. Written informed consent was obtained prior to inclusion in the study and in Summer 2018, 21 interviews were conducted in a private room in the clinic by HI.

### Interviews

Seven experts by experience expressed their preference for semi structured one-to-one interviews to discuss their health concerns, which are a suitable feminist data collection method to enable detailed exploration of personal lived experiences and ability to build a close relationship with participants which can lead to richer, more detailed data (Finch, 1984). A further ten experts by experience guided the development of the questions by identifying the domains of exercise, environment, illness/disease and house as aspects of health they felt women would have the most concerns around. These domains were displayed as cartoon sketches to facilitate discussion during the interviews. An interview guide (see Table 1) was developed by HI and MC. The guide used mainly open-ended questions to facilitate discussions. Two other interview guides used in qualitative research with South Asian populations were examined to ascertain the most appropriate terminology to adopt (Brown et al., 2006; Manikam et al., 2017). Five experts by experience reviewed the interview topic guide and further to their suggestions, the guide was iteratively refined. The interview guide was pilot tested on three experts by experience. The findings from the pilot have not been included in the data for this study. Interviews were conducted in person, solely by HI. Fieldnotes were captured in a journal immediately after the interview. Interviews were audio-recorded and lasted between 20–40 min.

**Table 1:** Interview topic guide

1. Are there any important things you think we should look at to help make things better for you and your family?
2. What are your concerns/worries about healthy eating and exercise for you and your family? (Probed for: weddings, Eid, Ramadan, visiting guests, gym, swimming, going for walks)
3. Do you feel that there is enough information and support available for you to be healthy?
4. What help and support is available for you to live a healthy lifestyle?
5. Is there anything else you would like to talk about?

### Data Analysis

Interviews were transcribed verbatim by HI and anonymised by removing names. Inductive thematic analysis utilizing six steps (Braun & Clarke, 2006) was undertaken by HI. This involved line by line coding of 21 transcripts during ongoing data collection to develop an initial coding scheme. A codebook was then created and subsequently refined several times throughout the process by the research team. All authors worked together to create the main themes from the codes. Three representative transcripts were closed-coded by MC to strengthen credibility. Although it is good practice in qualitative research to return transcripts to participants for member checking to verify their accuracy (Loeb & Steffensmeier, 2011), it was not possible to do this due to participants being part of a pre-existing study (BIBBS) and access to them before and after the study was limited.

### Researcher positionality

It was essential that the researchers questioned their own assumptions as is propagated by feminist research as we played a crucial role in data collection and interpretation. The research team are PhD level qualified with expertise in qualitative research in the UK. HI is a British Pakistani Muslim living in Bradford and has an interest in health inequalities faced by South Asian, Muslim women. The other authors are white British women. JW’s research interests include geographical, income and ethnic inequalities and their impact on child health, size and growth. RM’s research focuses on improving the health of families and reducing inequalities. MC’s research interests are ethnic health inequalities (including migrant) in communities. Our different identities and previous knowledge allowed us to unpick the data and question one another’s interpretations.

## Findings

**Fig. 1:**
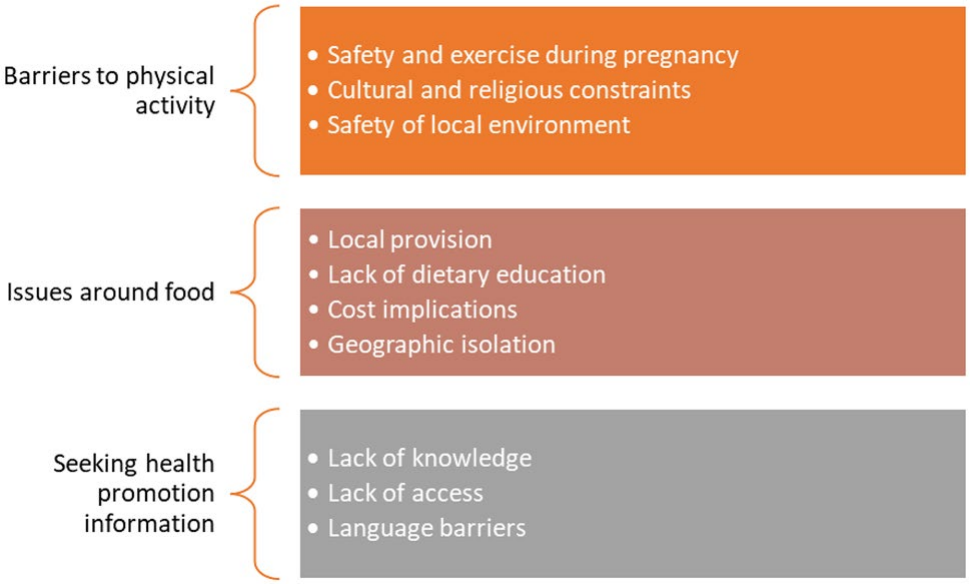
Themes and codes

Three key themes developed from the data: (1) barriers to physical activity (2) issues around food, and (3) seeking health promotion information. Direct quotes are used to support the analysis, with participants’ numbers to protect anonymity. See Fig. 1 for themes.

### Barriers to Physical Activity

Most participants expressed a strong desire to be physically active outdoors while pregnant but highlighted a range of barriers which prevented this. These included concerns around the safety of exercising during pregnancy, with some women stating they felt it would increase their chance of injury. Consequently, they stopped engaging in organized sport and physical activity: “I was going to the gym regularly until I got pregnant so now it’s not safe. I’ll start again after baby” (p7). Other women felt that even gentle physical activities were unsafe: “I will start after baby. I don’t feel it’s safe to go for walks yet.” (p1).

Although walking outdoors appeared to be a preferable form of physical activity, there was consensus amongst participants that they felt uncomfortable engaging in this while residing in densely populated South Asian areas. They cited feeling shame and embarrassment in response to people in the neighbourhood gossiping about them as walking outdoors was considered culturally taboo in the community in some areas, especially those with a higher-than-average concentration of Pakistani residents: “There is a park, yeah. I would not go though. Asian area and too many people talking.” (p4*).*

Some women overcame this by driving away from their areas and then walking for physical activity, where they were not recognised: “I don’t like to go for walks in my area so I drive into town and then walk around town so I would never walk around my area.” (p9).

Women discussed other safety concerns which prevented them and their children walking in the local parks. This included drug dealing in daylight, the high number of young male teens and adult men loitering in the park on the streets, the presence of people consuming alcohol, exposure to drug needles, and the amount of litter in the area:

*Taking him (child) to the park and there’s rubbish on the floor. Cans, bottles, rubbish. He runs towards things and we know he should not so that affects taking him out on a daily basis to get our exercise.* (p17).

Many participants discussed undertaking physical activity indoors and barriers to this. One reason for not engaging in indoor physical activity such as attending the gym when not pregnant, was the lack of female-only swimming classes or female-only exercise classes/gyms: “The other thing is that the gyms are mostly mixed, and I don’t want to exercise and turn around to see my male neighbour exercising next to me. So embarrassing.” (p19).

Others cited religious reasons as a barrier to indoor physical activity engagement, highlighting a lack of facilities that were compatible with their Islamic faith: “If there were facilities for purdah (observation of religious attire) ladies, there must have been a way that I would have come out and done it.” (p12).

### Issues Around Food

Participants discussed difficulties in consuming a healthy diet. This included the heavy presence of local fast-food outlets in their neighbourhood selling unhealthy, cheap food rather than healthy food options: “You only get unhealthy pizza and burger places in my area. They don’t sell anything healthy in these take-outs.” (p6).

Consequently, women felt it was more difficult to promote healthy eating in their household. They remarked that when they had prepared healthy, home-cooked meals, family members purchased calorific food from local takeaways, which meant that home-cooked food often resulted in waste: “I made vegetable curry last time, trying to be healthy, and my son walked in with huge boxes of chicken parmesan. No one ate what I made and I had to throw it out a couple of days later.” (p16).

Due to the lack of healthy food locally, women discussed having to travel to other areas of Bradford to access healthier foods in restaurants and takeaways. Many participants, however, mentioned that even when there were healthy food options in local takeaways, their financial situation meant that they struggled to eat healthily and they consumed inexpensive, unhealthy food rather than healthy, costly food: “Obviously, I can feed us takeout on a fiver, rather than spend a lot more on healthy food they have there that I can’t really afford.” (p15).

Another difficulty with eating healthily was confusion about recommended portion sizes. Women were generally aware of the advised portion guidelines for widely consumed food such as rice and meat, however, they struggled to determine how this equated with traditional Asian food that were regularly consumed as part of their daily diet, in pregnancy and otherwise, such as chapattis and curries: “There should be more help with portion sizes for Asian food and just food generally, just to make you more familiar.” (p5).

This lack of information meant that many women guessed the correct portion size of commonly consumed Asian food they had prepared, admitting their estimations were likely to be incorrect.

### Seeking Health Promotion Information

Despite being pregnant and having a midwife, there was concern amongst some participants about the lack of information and support available to them on maintaining healthy lifestyles. This included accessing information about consuming a healthy diet: “I don’t know where to go to get information on what a healthy diet is.” (p8).

For some women, the internet was cited as the main place to seek information on how to be healthy due to this lack of knowledge: “I would look on the internet, I actually don’t know where else to ask.” (p1) Some participants received support from their gym, their family doctor (GP), Sure Start centres, and organisations where they worked “I would go to the Dr to find out if I wanted information on how to eat healthy and exercise.” (p9) However, other participants believed that although the GP was the best person to provide advice, they had difficulty making GP appointments:

*The GP should give advice on healthy eating and being healthy, but I don’t even bother with them as I’ll be on the phone with them for ages to get an appointment and don’t even get it in the end so what’s the point even trying?* (p12).

Participants believed language barriers led to a lack of knowledge and understanding about health promotion messages by some Pakistani women:

*I have family members who can’t speak English and they won’t speak to any professional about their health. I can’t even see them enquiring about how to live a healthy lifestyle so aside from me telling them what they should eat and how to move, they wouldn’t know what healthy was. It’s not as though they can go online and research things like we can*. (p18).

## Discussion

The aim of this study was to explore the health concerns of pregnant Pakistani women living in deprived inner-city areas of Bradford. The three themes identified provide insight into some of the issues encountered by this population, preventing them from adopting a healthy lifestyle during their pregnancy. These findings have implications for researchers, policy makers, midwives and other health practitioners with an interest in improving the health of Pakistani women living in Bradford, of childbearing age.

Interestingly, despite the women in the present study being pregnant, pregnancy related concerns were made explicit only in relation to safety and physical activity. There is a lack of research on the physical activity barriers encountered by pregnant British Pakistani women, as well as a lack of information on their level of physical activity. National guidance in the UK from the Department of Health and Social Care (2019) recommends a minimum of 150 min of moderate intensity physical activity throughout pregnancy yet it was clear that the women in this study were not achieving this target, with misconceptions surrounding safety in physical activity cited as a major reason for their inactivity. Efforts aimed at increasing the uptake of physical activity in this population should focus on delivering education to address misconceptions around safety and exercise in pregnancy. Healthcare providers, midwives, and health promotion practitioners need to provide more detailed guidance on the safety of physical activity for their patients in this population. Given the frequent interactions between midwives and pregnant women, targeted education by the midwife, in particular, is needed to allay fears around safety.

The finding that Pakistani women did not attend physical activity facilities due to their lack of cultural appropriateness is in line with previous literature (Iqbal et al., 2022). According to Jepson et al. (2012), swimming and using the gym are two of the most popular activities for UK residents, but many South Asian women report cultural barriers which means that they are unable to make use of them. To overcome this issue, it has been suggested that walking to increase physical activity levels is most suited to South Asian communities (Cross-Bardell et al., 2015). This does not, however, address the issue of Pakistani women desiring to participate in these physical activities yet being unable to due to cultural barriers. Providing women-only exercise classes and facilities in settings familiar and trusted to women such as community centres and similar settings, could increase the uptake of physical activity in this group.

British Pakistani women report feeling uncomfortable walking alone and mention the cultural inappropriateness for women to be active (Lawton et al., 2006). In keeping with the literature, women in this study discussed cultural taboos and feelings of shame and embarrassment associated with walking outdoors (Patel et al., 2012). This is significant given that research has shown that high levels of physical activity in mothers results in increased levels of physical activity in children (Hesketh et al., 2019). When designing interventions to increase physical activity uptake in this population, it is important to acknowledge that cultural beliefs such as these are deeply engrained and are internalised by many Pakistani women and as such, there may not be an easy solution to overcome these. More research is required exploring effective, culturally appropriate ways to increase physical activity among this population.

Feeling unsafe acted as a deterrent to engaging in physical activity, which included visiting greenspaces such as parks. Issues of personal safety have been identified as a barrier to South Asian people undertaking physical activity in other studies (Cross-Bardell et al., 2015). Research has shown that low income, multi-ethnic populations in deprived areas experience similar barriers to accessing greenspaces around fear of crime, antisocial behaviour, and fear of accidents (Cronin-de-Chavez et al., 2019). Living near, and spending time in greenspace can not only improve physical and mental wellbeing by reducing depressive symptoms (McEachan et al., 2016) but can also improve pregnancy outcomes (Laurent et al., 2013), especially in lower socio-economic groups (Dadvand et al., 2012). A policy aim should be to address the structural and infrastructural conditions of deprived areas to increase the safety of the outdoor environment. This may increase the uptake of walking in local neighbourhoods.

Women were especially concerned about the high number of unhealthy fast-food outlets in their area and the impact this had on their and their family’s health. The most deprived areas of the UK have five times as many fast-food outlets as the most affluent areas and Bradford was identified as home to the tenth most unhealthy British High street (Wise, 2018). Policies aimed at restricting access to unhealthy food eateries, lowering the cost of healthy food, and incentivising healthy food retailers to locate in economically deprived areas are strategies that warrant consideration to increase health equity.

Like previous research, we found that women desired more knowledge on how to live a healthy lifestyle (Ludwig et al., 2011) yet they were uncertain of where to obtain health promotion knowledge. During pregnancy, much of the role of the midwife is in health promotion yet despite this, women felt that this was their general practitioner’s role. Primary health care professionals’ advice and support has been identified as a motivator in the initiation of exercise and physical activity in South Asians more generally (Horne & Tierney, 2012). It is important to acknowledge, however, that GP services may not be the most appropriate avenue. There is a need to publicise services offering health promotion advice that is accessible by Pakistani women. In pregnancy, this education should be delivered by midwives who have huge scope to provide an educational experience during the various interactions they have with pregnant women.

### Strengths and Limitations

To our knowledge, this is the first study that explores the health concerns of pregnant Pakistani women residing in the UK. The qualitative methodology offered a broad insight into women’s experiences, which is important due to the disproportionate poor health outcomes faced by British Pakistani women (Garcia et al., 2017; Insan et al., 2022). A strength to this study was the insider status of the main author who conducted the interviews. Possessing similar characteristics to the participants can assist significantly in rapport building, sharing collective experiences and discussing common features of their lives (Iqbal et al., 2023). This enabled an atmosphere of trust which may have increased the reliability of the findings. Care was taken, however, to diminish any bias that may have emanated from the main authors insider status. This included asking for clarification when participants assumed HI knew what was mean by something because of their shared characteristics.

Non-english-speaking women were not included in the sample due to time constraints. The health concerns identified in this study, therefore, may not reflect a range of experiences. Future research would benefit from obtaining perspectives from a wider demographic. In addition, the location in which the interview was conducted i.e., a clinical setting, may have influenced women’s level of comfort compared to home and the quality of their responses.

## Conclusion

British pregnant Pakistani women living in deprived areas identified health concerns relating to physical activity barriers, issues surrounding food and seeking health promotion information, all of which prevent them from being healthy before embarking on pregnancy. Researchers, midwives and other health professionals, local authority and policy makers interested in improving the health of pregnant South Asian women living in deprived areas may use these findings to develop further research in this area and interventions to improve health outcomes for South Asian women and their children.

## Data Availability

The data that support the findings of this study are available from the corresponding author upon reasonable request.
